# Supplementary material for: Long-range Cooper pair splitter with high entanglement production rate
Source: Sci Rep. 2015 Jan 5;5:7607. doi: 10.1038/srep07607 (PMC5154587; doi:10.1038/srep07607)
Supplement: Supplementary Information — for Long-range Cooper pair splitter with high entanglement production rate [file srep07607-s1.pdf]

Supplementary information for "Long-range Cooper pair splitter with high entanglement production rate"

Wei Chen,[1,*] D. N. Shi,[1] and D. Y. Xing[2]

[1]*College of Science, Nanjing University of Aeronautics and Astronautics, Nanjing 210016, China*

[2]*National Laboratory of Solid State Microstructures and Department of Physics, Nanjing University, Nanjing 210093, China*

PARTICLE-HOLE SYMMETRY OF THE SCATTERING MATRIX

We deduce the relation between the scattering amplitudes for the electron and hole incident cases. The second quantized Bogoliubov-de Gennes Hamiltonian is

$$\hat{H} = \frac{1}{2}\int dx \Phi^\dagger(x)\mathcal{H}(x)\Phi(x), \tag{S.1}$$

where the $\mathcal{H}(x)$ is defined by Eq. (1) in the main text. The Nambu representation is $\Phi = (\hat{\psi}_\uparrow, \hat{\psi}_\downarrow, \hat{\psi}^\dagger_\uparrow, \hat{\psi}^\dagger_\downarrow)^\mathrm{T}$ with "T" representing a matrix transpose. The four-component operator Φ satisfies $(\Phi^\dagger)^\mathrm{T} = \tau_x\Phi$ with τ_x the Pauli matrix operating on the particle-hole components. By applying this restriction on Eq. (S.1), one obtains the particle-hole transformation invariance of the Hamiltonian as

$$\mathcal{H} = -\Xi\mathcal{H}\Xi^{-1}, \tag{S.2}$$

where $\Xi = \tau_x K$ is the particle-hole transformation operator and K represents the complex conjugate. As a result, if ψ is an eigenstate of $\mathcal{H}$ with energy E as

$$\mathcal{H}\psi(E) = E\psi(E), \tag{S.3}$$

then we obtain

$$\mathcal{H}\Xi\psi(E) = -E\Xi\psi(E), \tag{S.4}$$

by using Eq. (S.2). This means $\Xi\psi(E) = \psi(-E)$ is an eigenstate of $\mathcal{H}$ as well with energy $-E$.

Next we prove that the particle-hole symmetry of the Hamiltonian $\mathcal{H}$ results in the particle-hole symmetry of the scattering matrix. For the translation invariant system, the eigenstate is a plane wave as

$$\psi_{\eta\sigma}(k,E) = (u_\uparrow, u_\downarrow, v_\downarrow, v_\uparrow)^\mathrm{T} e^{i(kx-Et/\hbar)}, \tag{S.5}$$

where η, σ are the particle-hole and spin indexes, respectively. Performing the particle-hole transformation on the wave function leads to

$$\begin{aligned}\Xi\psi_{\eta\sigma}(k,E) &= (v^*_\downarrow, v^*_\uparrow, u^*_\uparrow, u^*_\downarrow)^\mathrm{T} e^{-i(kx-Et/\hbar)}\\ &= \psi_{\bar{\eta}\bar{\sigma}}(-k,-E),\end{aligned} \tag{S.6}$$

which means the particle-hole transformation results in a flip of both particle-hole and spin components. Importantly, the moving direction of the quasiparticle is invariant after the particle-hole transformation, for the momentum and energy change their signs simultaneously. Therefore, an incident (outgoing) wave remains an incident (outgoing) wave after the particle-hole transformation.

In general, the incident and outgoing waves can be expressed as

$$\begin{aligned}|\Psi^\mathrm{i}\rangle &= \sum_{\alpha\eta\sigma} \tilde{a}_{\alpha\eta\sigma}(E)\psi^\mathrm{i}_{\alpha\eta\sigma}(E)\\ |\Psi^\mathrm{o}\rangle &= \sum_{\alpha'\eta'\sigma'} \tilde{b}_{\alpha'\eta'\sigma'}(E)\psi^\mathrm{o}_{\alpha'\eta'\sigma'}(E),\end{aligned} \tag{S.7}$$

where $\tilde{a}_{\alpha\eta\sigma}(E), \tilde{b}_{\alpha'\eta'\sigma'}(E)$ are the wave amplitudes of the incident and outgoing waves. We have omitted the momentum index, for its sign corresponds to the incident or outgoing waves. The amplitudes of the incident and outgoing waves are related by the scattering matrix as

$$\tilde{b}_{\alpha'\eta'\sigma'}(E) = S^{\alpha'\alpha}_{\eta'\eta,\sigma'\sigma}(E)\tilde{a}_{\alpha\eta\sigma}(E), \tag{S.8}$$

which can be obtained by solving the Bogoliubov-de Gennes equation. $S^{\alpha'\alpha}_{\eta'\eta,\sigma'\sigma}(E)$ is the scattering amplitude for an incident wave in the α lead with a particle-hole component η and spin σ being scattered into an outgoing wave in the α' lead with a particle-hole component η' and spin σ'.

Performing the particle-hole transformation on the wave functions (S.7) results in

$$\begin{aligned}\Xi|\Psi^{\rm i}\rangle &= \sum_{\alpha\eta\sigma} \tilde{a}^*_{\alpha\eta\sigma}(E)\psi^{\rm i}_{\alpha\bar{\eta}\bar{\sigma}}(-E)\\ \Xi|\Psi^{\rm o}\rangle &= \sum_{\alpha'\eta'\sigma'} \tilde{b}^*_{\alpha'\eta'\sigma'}(E)\psi^{\rm o}_{\alpha'\bar{\eta}'\bar{\sigma}'}(-E).\end{aligned} \tag{S.9}$$

Since the above wave functions still satisfy the same Bogoliubov-de Gennes equation, the amplitudes of the incident and outgoing waves are also related by the same scattering matrix as

$$\tilde{b}^*_{\alpha'\eta'\sigma'}(E) = S^{\alpha'\alpha}_{\bar{\eta}'\bar{\eta},\bar{\sigma}'\bar{\sigma}}(-E)\tilde{a}^*_{\alpha\eta\sigma}(E). \tag{S.10}$$

Note that the sign of the energy in the matrix elements is reversed, for the wave functions after transformation possess an energy of $-E$. Comparing Eq. (S.8) and Eq. (S.10), we arrive at the particle-hole symmetry of the matrix as

$$S^{\alpha'\alpha}_{\eta'\eta,\sigma'\sigma}(E) = S^{\alpha'\alpha *}_{\bar{\eta}'\bar{\eta},\bar{\sigma}'\bar{\sigma}}(-E), \tag{S.11}$$

which means that the amplitude for a spin-up (spin-down) incident electron (hole) with an energy E being scattered into a spin-up (spin-down) outgoing electron (hole) equals the amplitude for a spin-down (spin-up) incident hole (electron) with an energy $-E$ being scattered into a spin-down (spin-up) outgoing hole (electron).

Specifically, by adopting the notation of the scattering amplitudes in the main text, which correspond to the matrix elements through $a_1(E) = S^{LL}_{he,\uparrow\uparrow}(E), a_2(E) = S^{RL}_{he,\uparrow\uparrow}(E), b_1(E) = S^{LL}_{ee,\uparrow\uparrow}(E), b_2(E) = S^{RL}_{ee,\uparrow\uparrow}(E)$ and $a^h_{1\sigma}(E) = S^{LL}_{eh,\sigma\sigma}(E), a^h_{2\sigma}(E) = S^{RL}_{eh,\sigma\sigma}(E), b^h_{1\sigma}(E) = S^{LL}_{hh,\sigma\sigma}(E), b^h_{2\sigma}(E) = S^{RL}_{hh,\sigma\sigma}(E)$, and utilizing the relations $S^{\alpha'\alpha}_{\bar{\eta}\eta,\sigma\sigma}(E) = -S^{\alpha'\alpha}_{\bar{\eta}\eta,\bar{\sigma}\bar{\sigma}}(E), S^{\alpha'\alpha}_{\eta\eta,\sigma\sigma}(E) = S^{\alpha'\alpha}_{\eta\eta,\bar{\sigma}\bar{\sigma}}(E)$, we obtain

$$\begin{aligned}a^h_{1\uparrow}(E) &= -a^h_{1\downarrow}(E) = -a^*_1(-E)\\ a^h_{2\uparrow}(E) &= -a^h_{2\downarrow}(E) = -a^*_2(-E)\\ b^h_{1\uparrow}(E) &= b^h_{1\downarrow}(E) = b^*_1(-E)\\ b^h_{2\uparrow}(E) &= b^h_{2\downarrow}(E) = b^*_2(-E).\end{aligned} \tag{S.12}$$

DERIVATION OF THE ENTANGLED STATES

In order to obtain the entangled states generated via the inverse CAR, we start with the many-body incident state of $|\Psi_{\rm in}\rangle = \prod_{0<E<|eV|} \gamma^{\rm i\dagger}_{L\uparrow,E}\gamma^{\rm i\dagger}_{L\downarrow,E}|0\rangle$, where the incident holes occupy the energy window from the Fermi level to $|eV|$ in the left N region. The many-body outgoing wave can be obtained by expressing the operators of the incident wave by that of the outgoing wave, i.e., Eq. (4) in the main text, which leads to

$$|\Psi_{\rm out}\rangle = \prod_{0<E<|eV|,\sigma} \left(b^h_{1\sigma}(E)\gamma^{\rm o\dagger}_{L\sigma,E} + a^h_{1\sigma}(E)c^{\rm o\dagger}_{L\sigma,E} + b^h_{2\sigma}(E)\gamma^{\rm o\dagger}_{R\sigma,E} + a^h_{2\sigma}(E)c^{\rm o\dagger}_{R\sigma,E}\right)|0\rangle \tag{S.13}$$

Then we introduce a new vacuum state $|\tilde{0}\rangle$, which is related to the original one through $|0\rangle = \prod_{eV<E<0} c^{\rm o\dagger}_{L\uparrow,E}c^{\rm o\dagger}_{L\downarrow,E}|\tilde{0}\rangle$. Inserting this equality into the above equation results in

$$\begin{aligned}|\Psi_{\rm out}\rangle &= \prod_{0<E<|eV|,\sigma} \left(b^h_{1\sigma}(E)\gamma^{\rm o\dagger}_{L\sigma,E} + a^h_{1\sigma}(E)c^{\rm o\dagger}_{L\sigma,E} + b^h_{2\sigma}(E)\gamma^{\rm o\dagger}_{R\sigma,E} + a^h_{2\sigma}(E)c^{\rm o\dagger}_{R\sigma,E}\right) \prod_{eV<E'<0,\sigma'} c^{\rm o\dagger}_{L\sigma',E'}|\tilde{0}\rangle\\ &= \prod_{0<E<|eV|,\sigma} \left(b^h_{1\sigma}(E)\gamma^{\rm o\dagger}_{L\sigma,E} + a^h_{1\sigma}(E)c^{\rm o\dagger}_{L\sigma,E} + b^h_{2\sigma}(E)\gamma^{\rm o\dagger}_{R\sigma,E} + a^h_{2\sigma}(E)c^{\rm o\dagger}_{R\sigma,E}\right) c^{\rm o\dagger}_{L\bar{\sigma},-E}|\tilde{0}\rangle.\end{aligned} \tag{S.14}$$

By performing the particle-hole transformation $\gamma^{\mathrm{o}\dagger}_{\alpha\sigma,E} = c^{\mathrm{o}}_{\alpha\bar{\sigma},-E}$ and using the Eq. (S.12), we obtain

$$
\begin{aligned}
|\Psi_{\text{out}}\rangle &= \prod_{0<E<|eV|,\sigma} \Big(b^h_{1\sigma}(E)\mathbb{1} + a^h_{1\sigma}(E) c^{\mathrm{o}\dagger}_{L\sigma,E} c^{\mathrm{o}\dagger}_{L\bar{\sigma},-E} + b^h_{2\sigma}(E) c^{\mathrm{o}}_{R\bar{\sigma},-E} c^{\mathrm{o}\dagger}_{L\bar{\sigma},-E} + a^h_{2\sigma}(E) c^{\mathrm{o}\dagger}_{R\sigma,E} c^{\mathrm{o}\dagger}_{L\bar{\sigma},-E} \Big) |\tilde{0}\rangle \\
&= \prod_{0<E<|eV|} \big(\kappa|\mathcal{E}\rangle + \tilde{\kappa}|\tilde{\mathcal{E}}\rangle + |\mathcal{O}\rangle \big), \\
|\mathcal{E}\rangle &= \frac{\sqrt{2}}{2} \big(c^{\mathrm{o}\dagger}_{R\uparrow,E} c^{\mathrm{o}\dagger}_{L\downarrow,-E} - c^{\mathrm{o}\dagger}_{R\downarrow,E} c^{\mathrm{o}\dagger}_{L\uparrow,-E} \big)|\tilde{0}\rangle, \quad \kappa = -\sqrt{2} a^*_2(-E) b^*_1(-E), \\
|\tilde{\mathcal{E}}\rangle &= \frac{\sqrt{2}}{2} \big(c^{\mathrm{o}\dagger}_{R\uparrow,E} c^{\mathrm{o}\dagger}_{L\downarrow,E} - c^{\mathrm{o}\dagger}_{R\downarrow,E} c^{\mathrm{o}\dagger}_{L\uparrow,E} \big) c^{\mathrm{o}\dagger}_{L\downarrow,-E} c^{\mathrm{o}\dagger}_{L\uparrow,-E}|\tilde{0}\rangle, \quad \tilde{\kappa} = \sqrt{2} a^*_1(-E) a^*_2(-E),
\end{aligned}
\tag{S.15}
$$

where $|\mathcal{E}\rangle$ and $|\tilde{\mathcal{E}}\rangle$ are two kinds of nonlocal entangled states, and $|\mathcal{O}\rangle$ represents the other scattering states. For the optimal case $Z_2 = 0$, the EC process is completely suppressed in the CAR window $E \in \Delta(1 - i_s, 1 + i_s)$, so that $|\mathcal{O}\rangle$ reduces to

$$
\begin{aligned}
|\mathcal{O}\rangle &= b^{*2}_1(-E)|\tilde{0}\rangle - a^{*2}_1(-E) c^{\mathrm{o}\dagger}_{L\uparrow,E} c^{\mathrm{o}\dagger}_{L\downarrow,E} c^{\mathrm{o}\dagger}_{L\uparrow,-E} c^{\mathrm{o}\dagger}_{L\downarrow,-E}|\tilde{0}\rangle - a^{*2}_2(-E) c^{\mathrm{o}\dagger}_{R\uparrow,E} c^{\mathrm{o}\dagger}_{R\downarrow,E} c^{\mathrm{o}\dagger}_{L\uparrow,-E} c^{\mathrm{o}\dagger}_{L\downarrow,-E}|\tilde{0}\rangle - \sqrt{2} a^*_1(-E) b^*_1(-E)|\mathcal{E}\rangle_{\text{local}}, \\
|\mathcal{E}\rangle_{\text{local}} &= \frac{\sqrt{2}}{2} \big(c^{\mathrm{o}\dagger}_{L\uparrow,E} c^{\mathrm{o}\dagger}_{L\downarrow,-E} - c^{\mathrm{o}\dagger}_{L\downarrow,E} c^{\mathrm{o}\dagger}_{L\uparrow,-E} \big)|\tilde{0}\rangle.
\end{aligned}
\tag{S.16}
$$

The state $|\mathcal{O}\rangle$ is a superposition of the local entangled state $|\mathcal{E}\rangle_{\text{local}}$, the direct product states and the vacuum state. For the energy levels outside the CAR window or for the case of $Z_2 \neq 0$, more terms should be included in the state $|\mathcal{O}\rangle$.

---

* Electronic address: weichenphy@nuaa.edu.cn
